# Postoperative Complications of Beger Procedure

**DOI:** 10.1155/2015/970785

**Published:** 2015-08-24

**Authors:** Nayana Samejima Peternelli, Tali Wajsfeld, Felipe Henrique Yazawa Santos, Otavio Schmidt de Azevedo, Rodrigo Altenfelder Silva, Adhemar Monteiro Pacheco Junior

**Affiliations:** ^1^Santa Casa de Sao Paulo Medical School, Rua Cesario Mota Junior 112, 01221-020 São Paulo, SP, Brazil; ^2^Department of Surgery, Santa Casa de São Paulo Hospital, Rua Cesario Mota Junior 112, 01221-020 São Paulo, SP, Brazil

## Abstract

*Introduction*. Chronic pancreatitis (CP) is considered an inflammatory disease that may cause varying degrees of pancreatic dysfunction. Conservative and surgical treatment options are available depending on dysfunction severity. *Presentation of Case*. A 36-year-old male with history of heavy alcohol consumption and diagnosed CP underwent a duodenal-preserving pancreatic head resection (DPPHR or Beger procedure) after conservative treatment failure. Refractory pain was reported on follow-up three months after surgery and postoperative imaging uncovered stones within the main pancreatic duct and intestinal dilation. The patient was subsequently subjected to another surgical procedure and intraoperative findings included protein plugs within the main pancreatic duct and pancreaticojejunal anastomosis stricture. A V-shaped enlargement and main pancreatic duct dilation in addition to the reconstruction of the previous pancreaticojejunal anastomosis were performed. The patient recovered with no further postoperative complications in the follow-up at an outpatient clinic. *Discussion*. Main duct and pancreaticojejunal strictures are an unusual complication of the Beger procedure but were identified intraoperatively as the cause of patient's refractory pain and explained intraductal protein plugs accumulation. *Conclusion*. Patients that undergo Beger procedures should receive close outpatient clinical follow-up in order to guarantee postoperative conservative treatment success and therefore guarantee an early detection of postoperative complications.

## 1. Introduction

Chronic pancreatitis (CP) is a progressive inflammatory disease that may lead to exocrine and endocrine organ dysfunction [1]. An overwhelming 70% of cases result from alcohol abuse, 20% are idiopathic, and 10% result from other causes. Abdominal pain irradiating to the back is a frequently reported symptom. Pancreatic diabetes and fat malabsorption may also be present leading to both glucose intolerance and steatorrhea. Also, pain levels have been correlated to increased intraductal pressure, perineural inflammation, and severity of fibrosis especially in pancreas head [2]. Diagnosis is based on clinical findings and confirmed with imaging studies. Only 30% of radiographies detect pancreatic calcifications while ultrasound sensibility varies within 60–70% evidencing pancreatic enlargement and ductal dilatation. Conservative CP treatment focuses on management of pain and treatment of pancreatic insufficiency, which consists in alcohol intake and smoking cessation and fractionated diet adherence. Pancreatic enzyme intake associated with gastric acid suppression and analgesics consists in pharmacological adjuvant treatment. Although refractory chronic pain is considered the most common indication for surgical treatment, complications of CP such as pseudocyst formation, pancreatic and biliary ductal systems stricture, and suspected neoplasm may also require surgical intervention [3].

## 2. Case Presentation

**Figure 1:**
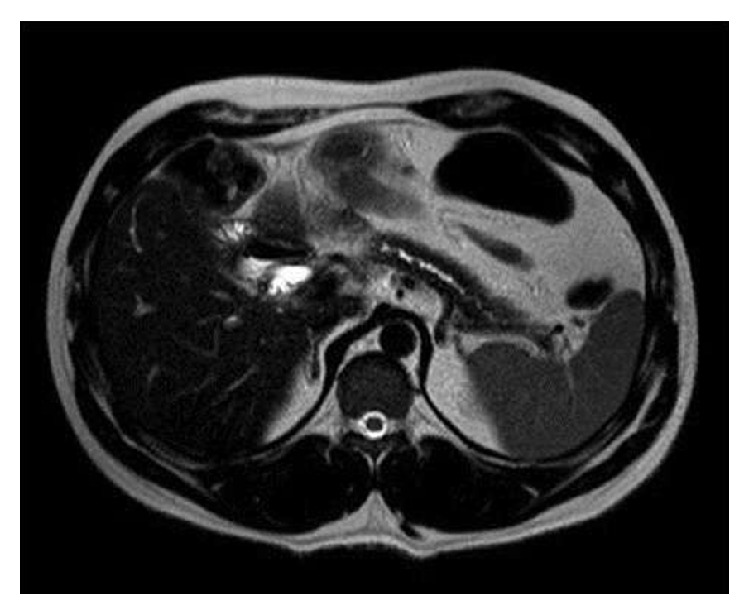
MRI shows main pancreatic duct dilation and stones.

A 36-year-old male was referred to an outpatient clinic due to constant chronic abdominal pain. The subject had a history of heavy alcohol intake and was diagnosed with CP. Initial conservative treatment included Omeprazole, Pancreatin, nonsteroidal analgesics, and lifestyle modifications that consisted in a fractionated diet and alcohol intake cessation. Despite conservative treatment, symptoms persisted with progressive weight loss and mild steatorrhea was reported. Initial CT scan showed small calcification areas on pancreatic head and sparse calcifications in pancreatic body and tail. Mild enlargement of the pancreatic head and fibrosis without defined focal lesions (longest axis 4.0 cm in its, normal up to 3 cm) was also present. Main pancreatic duct (maximum diameter of 1.0 cm) and common bile duct dilation were evidenced (maximum diameter of 1.4 cm). Following a 3-year conservative treatment failure, the patient underwent Beger procedure in January 2014 with no intraoperative complications and consisted in pancreatic head resection with main pancreatic duct stenotic portion responsible for main pancreatic duct dilation. Also, no further signs of intrinsic obstruction were found. The subject developed a biliodigestive anastomosis fistula as a postoperative complication that was conservatively treated and was discharged from the hospital. Three months after the Beger procedure, subject reported new onset epigastric and bilateral hypochondrial abdominal pain but no fever, vomiting, weight loss, or any signs of obstruction (including jaundice, diarrhea, dark urine, acholic stools, or itching). Subsequent laboratory findings excluded the possibility of biliary tract obstruction. A CT scan revealed diffuse thickening of small intestine and suggested inflammation near the residual pancreatic parenchyma. A subsequent MRI (Figure 1) evidenced pancreatic parenchymal abnormalities suggesting chronic pancreatic disease and two small contiguous collections in the cephalic portion of the pancreas were identified, the largest measuring 1.0 cm. In addition, a small dilatation of biliary tract was present (common bile duct measuring 0.8 cm and pancreatic duct measuring 0.3 cm in diameter). After clinical and imaging findings, diagnostic hypotheses were insufficient parenchymal resection of the pancreas head, persistent fistula inflammation, and pancreatic duct stenosis. Intestinal obstruction due to stricture of Roux-en-Y during the choledochojejunostomy was also suggested. Patient was readmitted within nine months and underwent new surgical approach. Intraoperative findings included main pancreatic duct and pancreaticojejunal anastomosis strictures. Pancreaticojejunal anastomosis was redone after extensive cleaning and protein plug removal. Dilatation of the main duct was performed, along with border debridement and V-shaped enlargement at the site of the previous anastomosis. Patient emerged with no postoperative complications, descending drain amylase levels, and no fever or wound infection, being discharged with follow-up in an outpatient clinic.

## 3. Discussion

Three surgical approaches have been described so far for chronic pancreatitis: decompression, pancreatic resection, and denervation. Some surgical procedures employ a combination of these approaches. Decompression therapies consist of duodenal-preserving pancreatic head resection (DPPHR) and local resection of the pancreatic head with extended longitudinal pancreaticojejunostomy (LR-LPJ), known as Beger and Frey procedures, respectively. Trials comparing Beger and Frey procedures have shown no statistical difference concerning morbidity, mortality, and pancreatic function, as both ensure gastrointestinal continuity. Frey procedure preserves the posterior capsule of the pancreas and requires pancreaticojejunal anastomosis, while DPPHR requires a pancreaticojejunal and a biliodigestive anastomosis. Therefore, surgical experience should be taken into consideration when electing which technique to perform [4]. Exocrine and endocrine dysfunction after Beger surgery progresses as an underlying CP and its course is minimally affected by the procedure. Nevertheless, according to Schlosser et al., improvement of pancreatic function has been observed [5]. Pain relief of 80–85% has been reported in a 5-year follow-up study and another 14-year follow-up study reported 78.8% of pain-free subjects. Diabetes incidence after DPPHR ranges within 8–21% and some patients show significant improvement in glucose metabolism and appears to be related to preservation of insulin and pancreatic polypeptide secretive function. DPPHR procedure complications include risk of duodenum ischemia related to inadequate perfusion of the posterior branch of the gastroduodenal artery. Increased surgical time must be considered, as pancreatic transection requires a tunnel anterior to the mesentericoportal vein (MPV) axis. Moreover, pancreaticojejunal anastomosis leaks may contribute to increased local inflammation [6].

## 4. Conclusion

During clinical investigations of refractory pain after Beger procedure, imaging may reveal worsened pancreas parenchymal abnormalities and protein plugs due to defective drainage of a narrowed main pancreatic duct or pancreaticojejunal anastomosis stricture. This case demonstrates an uncommon complication of Beger procedure. Therefore, patients that undergo surgical treatment have to be closely monitored in an outpatient clinic in order to guarantee postoperative conservative treatment and early detection of possible complications related to the initial surgical procedure.
